# DJ-1 interacts with the ectopic ATP-synthase in endothelial cells during acute ischemia and reperfusion

**DOI:** 10.1038/s41598-022-16998-3

**Published:** 2022-07-26

**Authors:** Alex Gallinat, Lina Badimon

**Affiliations:** 1 Cardiovascular Program-ICCC, IR-Hospital Santa Creu i Sant Pau, IIB-Sant Pau, c/Sant Antoni María Claret, 167, 08025 Barcelona, Spain; 2 Universitat Autònoma de Barcelona (UAB), Barcelona, Spain; 3 CIBERCV-Instituto de Salud Carlos III, Madrid, Spain; 4 UAB-Chair Cardiovascular Research, Barcelona, Spain

**Keywords:** Cell biology, Cell signalling, Stress signalling

## Abstract

Endothelial cells (ECs) play a central role in ischemia. ATP-Synthase is now recognized to be ectopically expressed in the cell surface of many cell types, with putative roles described in angiogenesis, proliferation, and intracellular pH regulation. DJ-1 is a multifunctional protein, involved in cell protection against ischemia, ischemia–reperfusion (I/R), and oxidative stress, that regulates mitochondrial ATP-synthase. Here we focused on the characterization of the endothelial dynamics of DJ-1, and its implication in the regulation of the ectopic ATP-synthase (ecATP-S) activity, during acute ischemia and I/R in ECs. We found that DJ-1 is secreted from ECs, by a mechanism enhanced in ischemia and I/R. A cleaved form of DJ-1 (DJ-1∆C) was found only in the secretome of ischemic cells. The ecATP-S activity increased following acute ischemia in ECs, coinciding with DJ-1 and DJ-1∆C secretion. The inhibition of DJ-1 expression inhibited the ecATP-S response to ischemia by ∼ 50%, and its exogenous administration maximized the effect, together with an enhanced Akt phosphorylation and angiotube-formation potential at reperfusion. Immunoprecipitation studies showed direct interaction between DJ-1 and the ecATP-S. Altogether suggesting that DJ-1 is actively cleaved and released from ischemic ECs and plays an important role in the regulation of the ecATP-S activity during acute ischemia and reperfusion.

## Introduction

Ischemia is defined as the stress resulting from the restriction of blood supply to a given tissue or organ. It is mainly caused by the capillary obstruction due to either microthrombus formation or microvascular damage. When this happens, oxygen unavailability disrupts the cellular metabolism leading to ATP depletion, acidosis, and the accumulation of detrimental products, ultimately resulting in an extensive cell death and organ dysfunction. Ischemia is a common hallmark of many diseases, as is the case of myocardial infarction, stroke, and kidney, limb, or intestinal ischemia, all of them responsible of a high morbidity and mortality worldwide. Furthermore, ischemia is also found in the core of most solid tumours, and is believed to play a role in the malignant cell progression^1^.

Endothelial cells (ECs), lining the circulatory system, play a pivotal role in regulating homeostasis and disease. Rather than a barrier, their key location between blood stream and the surrounding tissue, makes the endothelium an active player sensing and responding to hemodynamic changes and ischemic damage^2,3^. From normal embryonic development to tumour progression and the onset of an ischemic event, ECs provide multiple autocrine and paracrine signals supporting organ function^4^, and modulate the immune response^2^.

The F_O_F_1_ ATP-synthase is the enzyme responsible for the formation of ATP from ADP and inorganic phosphate, driven by the electrochemical gradient established through the electron transport chain in the mitochondria. Despite it was originally believed to exclusively locate in the inner mitochondrial membrane, its ectopic expression in the cell surface have been proven for a wide spectrum of cell types, including vascular ECs^5–7^, hepatocytes^8,9^, adipocytes^10^, lymphocytes^11^, keratinocytes^12^, muscle^13^, and neural cells^14,15^, in both tumour and normal conditions^16^. It is known to act as a receptor for angiostatin^6^, and apolipoprotein A-I^7,8^, and to promote tumour-recognition by the immune system^16–18^. In ECs, ectopic ATP-synthase (ecATP-S) have been recognized to play a role in angiogenesis, proliferation, and regulating intracellular pH^7,19,20^. Furthermore, its location within the lipid rafts and caveolae makes plausible a functional connection with purinoreceptors, modifying the local ATP/ADP concentrations, and thus promoting downstream signalling^9,11,21^. Since the ecATP-S highly increases its activity under acidic and hypoxic cell culture conditions^22^, a role in regulating cell function under ischemia could be presumed.

The early-onset Parkinson’s disease associated protein DJ-1 (also known as PARK7) is a protein with several pleiotropic functions including chaperone^23^ and protease^24^, deglycase^25,26^, transcriptional and translational regulator^27,28^, redox sensor^29^ and mitochondrial homeostasis keeper^30,31^, that has been shown to bind and regulate the mitochondrial F_O_F_1_ ATP-synthase activity^32^. A cleaved form of DJ-1, corresponding to a 15 carboxyl-terminal amino acids deletion (referred to as DJ-1∆C), has been suggested as the active form in in vitro testing, and cell protection effects have been proven^24,33,34^. Despite the exact role of DJ-1 and DJ-1∆C has not yet been elucidated, it is widely accepted to play a role in cell protection against ischemia, ischemia–reperfusion (I/R) and oxidative stress^35^. Here we have focused on the characterization of the endothelial dynamics of protein DJ-1 and DJ-1∆C, and their implication in the regulation of the ec-ATP-S activity, in a model of acute ischemia and I/R.

## Results

### Endothelial DJ-1 content declines during I/R

**Figure 1:**
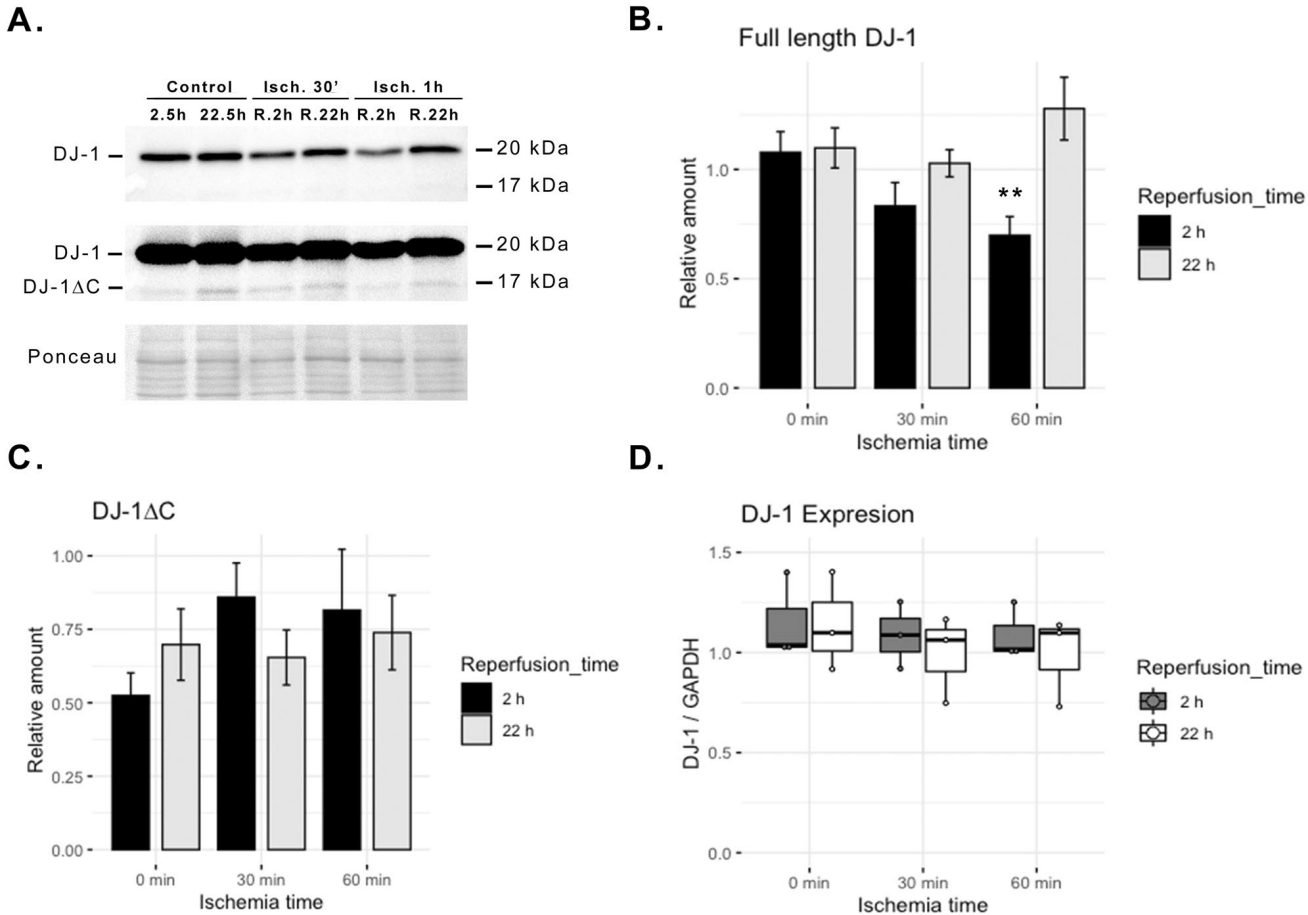
Endothelial DJ-1 content declines during I/R injury. ECs cultures were subjected to either 30 min or 1 h in vitro ischemia followed by 2 or 22 h of reperfusion, and both protein and RNA extracts were probed for DJ-1. (**A**) Representative western blot. (**B**) Quantification of endothelial full length DJ-1 upon I/R. (n = 5; **p < 0.01). (**C**) Quantification of endothelial DJ-1∆C upon I/R (n = 5).; and, (**D**) *park7* gene expression upon I/R in ECs (n = 3). Data presented as mean ± SEM. *ECs* endothelial cells, *I/R* ischemia–reperfusion. Corresponding uncropped western blot acquisitions can be found in Supplemental Fig. 2.

We investigated whether ECs subjected to I/R injury show a dysfunctional regulation of DJ-1 and/or its cleavage. Different periods of in vitro I/R were tested, and both the full-length and cleaved forms of DJ-1, as well as DJ-1 expression, were measured. A short period of reperfusion (i.e. 2 h) following ischemia led to a significant decline in intracellular full-length DJ-1 (*p* < 0.01; Fig. 1A–C). A significant effect was found in the interaction between duration of ischemia and duration of reperfusion (*p* < 0.05), meaning that DJ-1 decline at reperfusion was dependent on the severity of ischemia. Interestingly while after 2 h of reperfusion the levels of full-length DJ-1 were reduced from baseline (cells without ischemia), after 22 h of reperfusion, DJ-1 basal level was restored (Fig. 1A–C). DJ-1∆C was found intracellularly at much lower levels than DJ-1 and although these levels seemed to rise at reperfusion, differences did not reach significance at initially measured time-points (Fig. 1B,C). The assessment of DJ-1∆C dynamics at different reperfusion times after 1 h of ischemia revealed that while DJ-1∆C intracellular content falls during ischemia, it increases with time after reperfusion (Supplemental Fig. 1). Regardless of the effects of I/R on DJ-1 protein content, no differences in DJ-1 gene transcription were found (Fig. 1D).

### Acute ischemia and I/R promote DJ-1 and DJ-1∆C endothelial secretion

**Figure 2:**
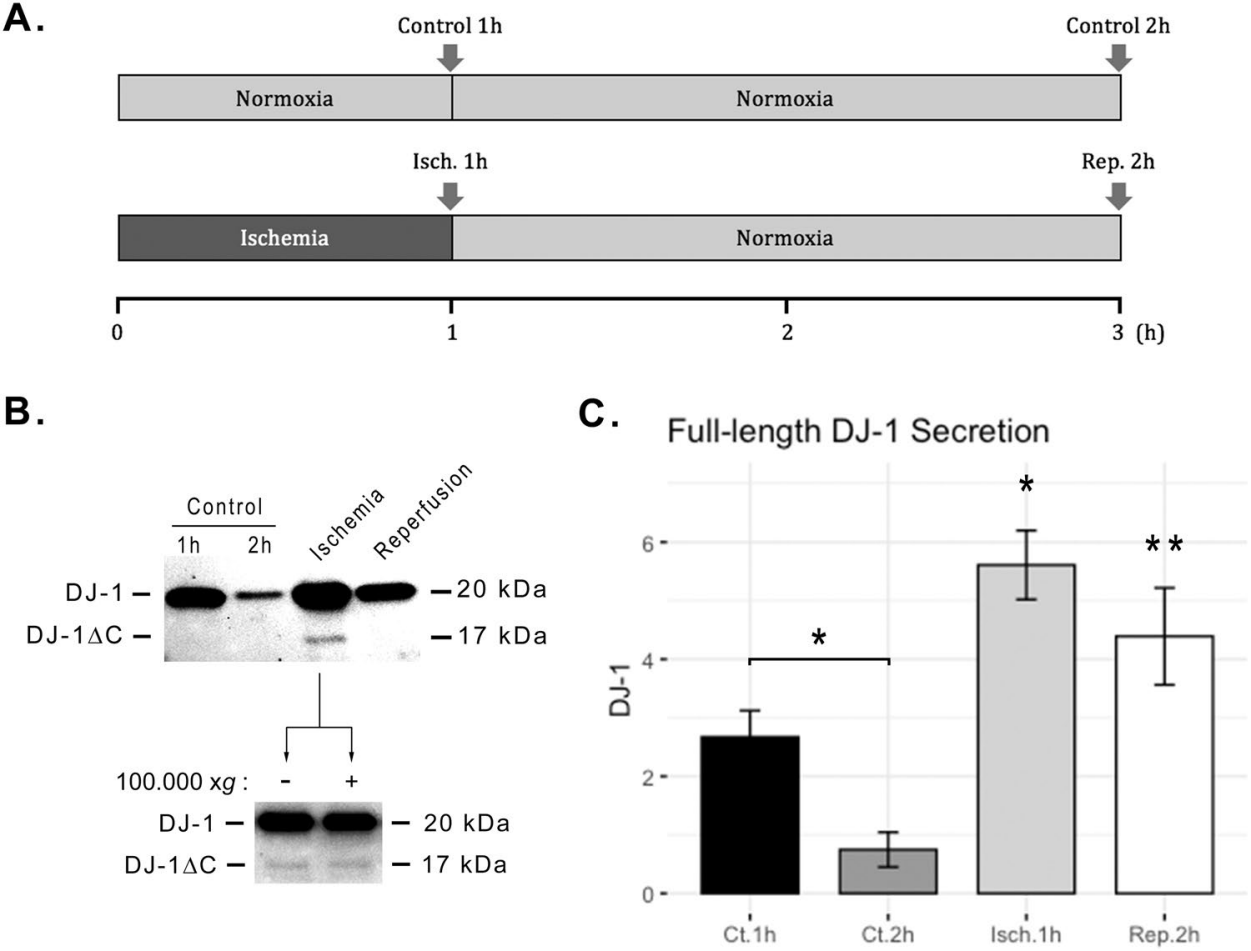
In vitro I/R promotes DJ-1 and DJ-1∆C endothelial secretion. ECs cultures were subjected to 1 h in vitro ischemia followed by 2 h of reperfusion, and supernatants from ischemia, reperfusion, and control cultures, were probed for DJ-1. (**A**) Timeline scheme representing the experimental work-flow and group definitions for the analysis of the secretome. Arrows represent supernatant collection for analysis and medium exchange. (**B**) Representative western blot. (**C**) Western blot quantification of secreted full length DJ-1 (n = 5; *p < 0.05; **p < 0.01; n.s., not significant). Data presented as mean ± SEM. *ECs* Endothelial cells. Corresponding uncropped western blot acquisition can be found in Supplemental Fig. 3.

The reduced levels of DJ-1 in ECs after I/R may be explained by its release from the cell. Thus, we analysed the presence of DJ-1 forms in the secretome of ECs subjected to I/R. Interestingly, the full-length form of DJ-1 was detectable in the cell secretome in each of the conditions tested, with a significantly higher abundance after ischemia (*p* < 0.05) and after I/R (*p* < 0.01; Fig. 2), suggesting an active release of DJ-1 by cells under ischemic stress. Moreover, DJ-1∆C was found to be present just in the secretome of cells during ischemia alone (Fig. 2B) and occasionally during reperfusion, but never in the controls. These results suggest that DJ-1 is cleaved into DJ-1∆C and actively released by ECs during ischemia. In order to check whether DJ-1 and DJ-1∆C are secreted in a soluble form or within extracellular vesicles (EVs), we removed EVs by ultracentrifugation (1 h at 100.000 × *g*) of the supernatants collected after 1 h of ischemia prior protein precipitation. There were no differences on DJ-1 and DJ-1∆C levels (Fig. 2B), indicating that both DJ-1 forms are found in the soluble secretome.

### DJ-1 down-regulation impairs ectopic ATP-synthase activity rise after ischemia

**Figure 3:**
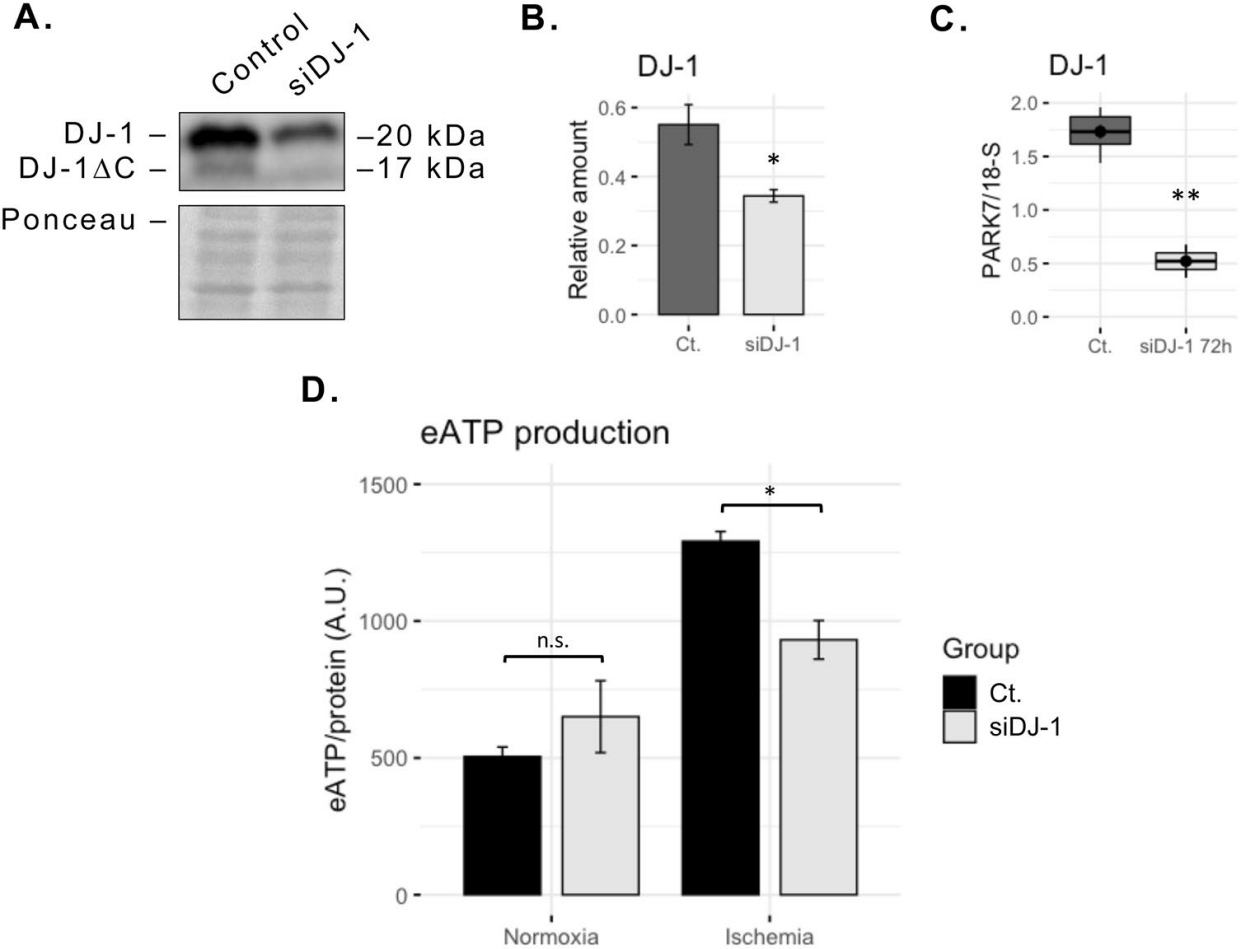
Extracellular ATP generation after ischemia is dependent on DJ-1. ECs cultures were transfected with a siRNA targeted to DJ-1, and subjected to 1 h in vitro ischemia. Extracellular ATP generation was then evaluated. (**A**) Representative western blot showing DJ-1 inhibition 72 h post-transfection. (**B**) Western blot quantification of DJ-1 72 h post-transfection (n = 4; *p < 0.05). (**C**) *park7* gene expression of control and knock-down cultures 72 h post-transfection (n = 3; **p < 0.01). (**D**) Extracellular ATP generation of DJ-1 knock-down and control cultures after 1 h in vitro ischemia compared to normoxic controls (n = 4; *p < 0.05; *n.s.* not significant). Data presented as mean ± SEM. *ECs* endothelial cells. Corresponding uncropped western blot acquisition can be found in Supplemental Fig. 4.

Given that DJ-1 has been previously reported to regulate the mitochondrial ATP-synthase activity^32^, and that ECs extracellular ATP generation increases after a period of incubation under ischemia-like conditions^22^, we sought to analyse whether ECs DJ-1/DJ-1∆C secretion under ischemia has an impact on the ecATP-S activity. Thus, we analysed the extracellular ATP generation of DJ-1 knocked-down ECs cultures exposed to normoxia and ischemia, compared to untransfected controls. After 1 h of ischemia, untransfected cultures exhibited nearly threefold increase in the extracellular ATP generation. Such increase after ischemia was significantly reduced in DJ-1 knocked-down cultures (*p* < 0.05; Fig. 3), indicating that protein DJ-1 is needed for the ecATP-S regulation after ischemia.

### Extracellular DJ-1 boosts ectopic ATP-synthase activity after ischemia

**Figure 4:**
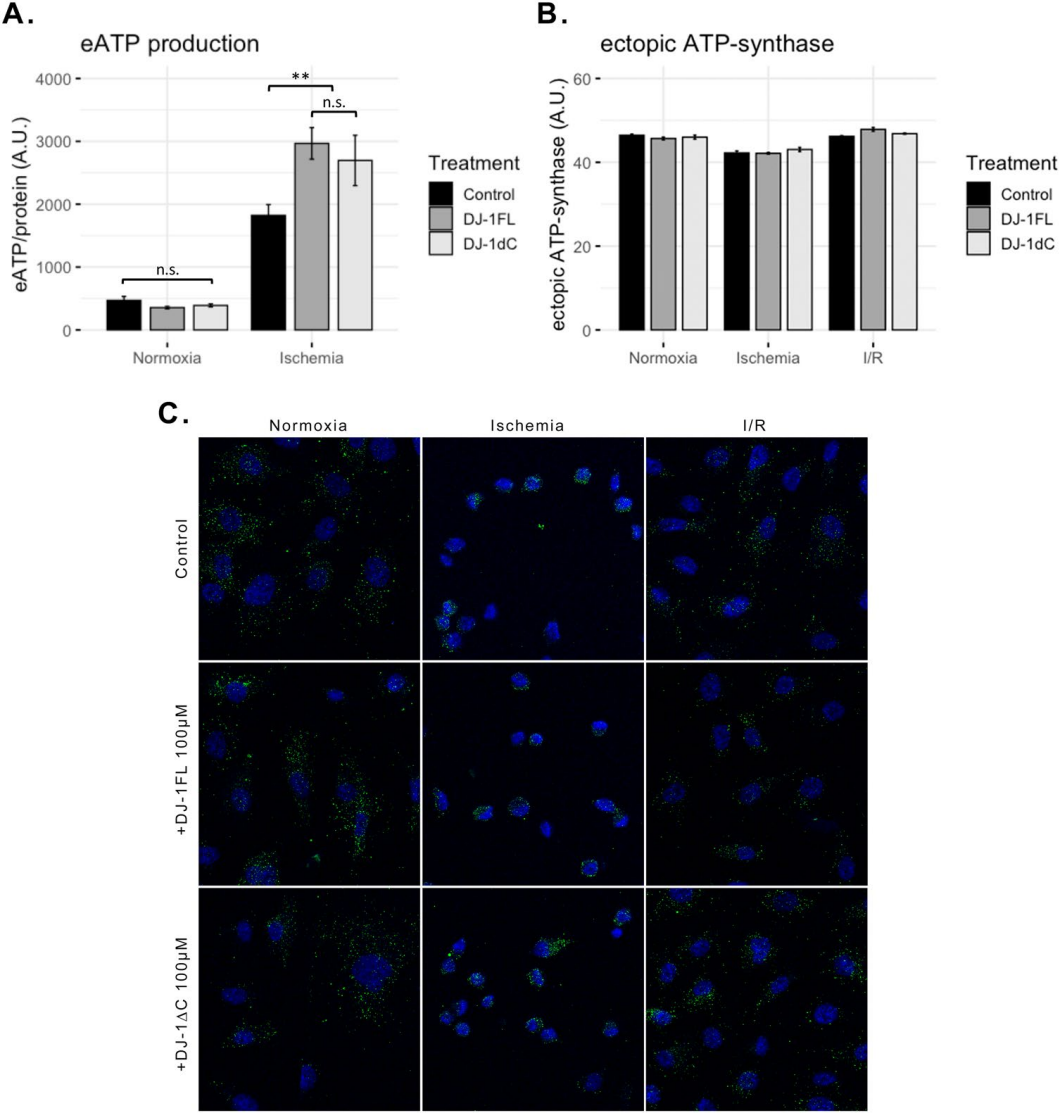
Extracellular DJ-1 promotes ectopic ATP-synthase activity following acute ischemia. ECs cultures were subjected to 1 h in vitro ischemia in the presence and the absence of full length DJ-1 and DJ-1∆C (100 nM), and both the extracellular ATP generation and the ecATP-S expression were evaluated. (**A**) Extracellular ATP generation after 1 h in vitro ischemia in the presence and the absence of full length DJ-1 and DJ-1∆C (n = 4; **p < 0.01; *n.s.* not significant). (**B**) ecATP-S expression measured by immunohistochemistry across conditions. (**C**) Representative images of the ecATP-S immunohistochemistry across conditions. Green signal corresponds to ecATP-S. *ECs* endothelial cells, *ecATP-S* ectopic ATP-synthase.

In order to test whether the reported effect of DJ-1 upon the activity of the cell surface ATP-synthase is dependent on the extracellular form, we tested the extracellular ATP generation in ECs cultures subjected to ischemia or normoxia, in the presence and the absence of extracellular recombinant full-length DJ-1 and DJ-1∆C (at 100 nM). The administration of DJ-1 resulted in a highly significant increase of the extracellular ATP generation after ischemia (*p* < 0.01), independently of the DJ-1 form employed (Fig. 4A). This effect could be explained either by an increase of the ecATP-S activity or localization to the cell surface. After measuring the ectopic expression of the ATP-synthase following ischemia and I/R in the presence and the absence of exogenous DJ-1 and DJ-1∆C, no differences were detected across conditions (Fig. 4B,C), meaning the reported changes in the extracellular ATP generation are a consequence of an activity increase rather than localization.

### Extracellular DJ-1 interacts with the ectopic ATP-synthase

**Figure 5:**
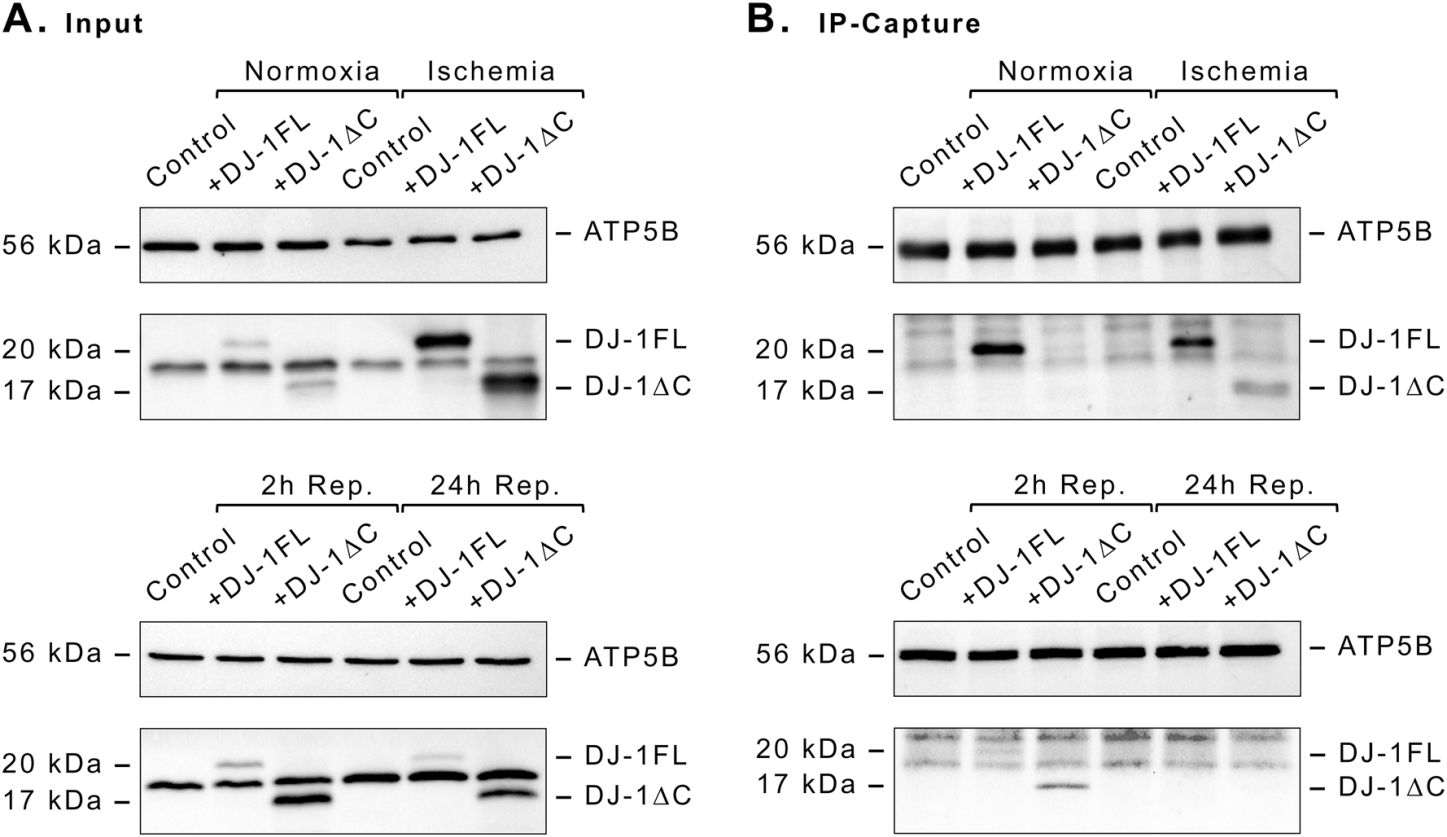
ATP-synthase co-immunoprecipitation with exogenous DJ-1 and DJ-1∆C in ischemia and I/R. ECs cultures were exposed to either 1 h ischemia or normoxia, in the presence and the absence of exogenously administrated DJ-1 or DJ-1∆C at 100 nM, with and without a reperfusion period of 2 h and 24 h (whithout exogenous DJ-1/DJ-1∆C). After indicated treatment, cells were thoroughly rinsed, lysed, and immunoprecipitated against ATP-synthase. (**A**) Western blot analysis of ATP synthase (ATP5B) and DJ-1/DJ-1∆C in whole cell lysates (immunoprecipitation input). (**B**) Western blot analysis of ATP synthase (ATP5B) and DJ-1/DJ-1∆C in the IP-captures. *ECs* endothelial cells, *IP* immunoprecipitate, *I/R* ischemia/reperfusion. Membranes were split in two before incubation. Corresponding uncropped western blot acquisitions can be found in Supplemental Fig. 5.

ECs were cultured for 1 h under ischemic or normoxic conditions, in the presence and the absence of exogenous DJ-1 and DJ-1∆C. Thereafter, cells were thoroughly rinsed, lysed, and immunoprecipitated against ATP-synthase. Immunocaptures were then assayed by western blot for both ATP-synthase and DJ-1. While exogenous full length DJ-1 was detectable in both normoxic and ischemic cell immunocaptures, interaction with DJ-1∆C was only found in cells under ischemia (Fig. 5A,B). In order to check whether DJ-1/DJ-1∆C association with the ATP-synthase is preserved over the course of reperfusion, the same experiment was performed in cultures subjected to ischemia and reperfusion in the presence and the absence of DJ-1 and DJ-1∆C during the ischemic stimuli. As a result, after 2 h of reperfusion some traces of DJ-1∆C remained detectable in the ATP-synthase immunocapture, while the full-length DJ-1 association with the ATP-synthase was lost. After 24 h of reperfusion, no association was detectable for DJ-1 of either form.

### Extracellular DJ-1 enhances Akt phosphorylation and angiotube formation

**Figure 6:**
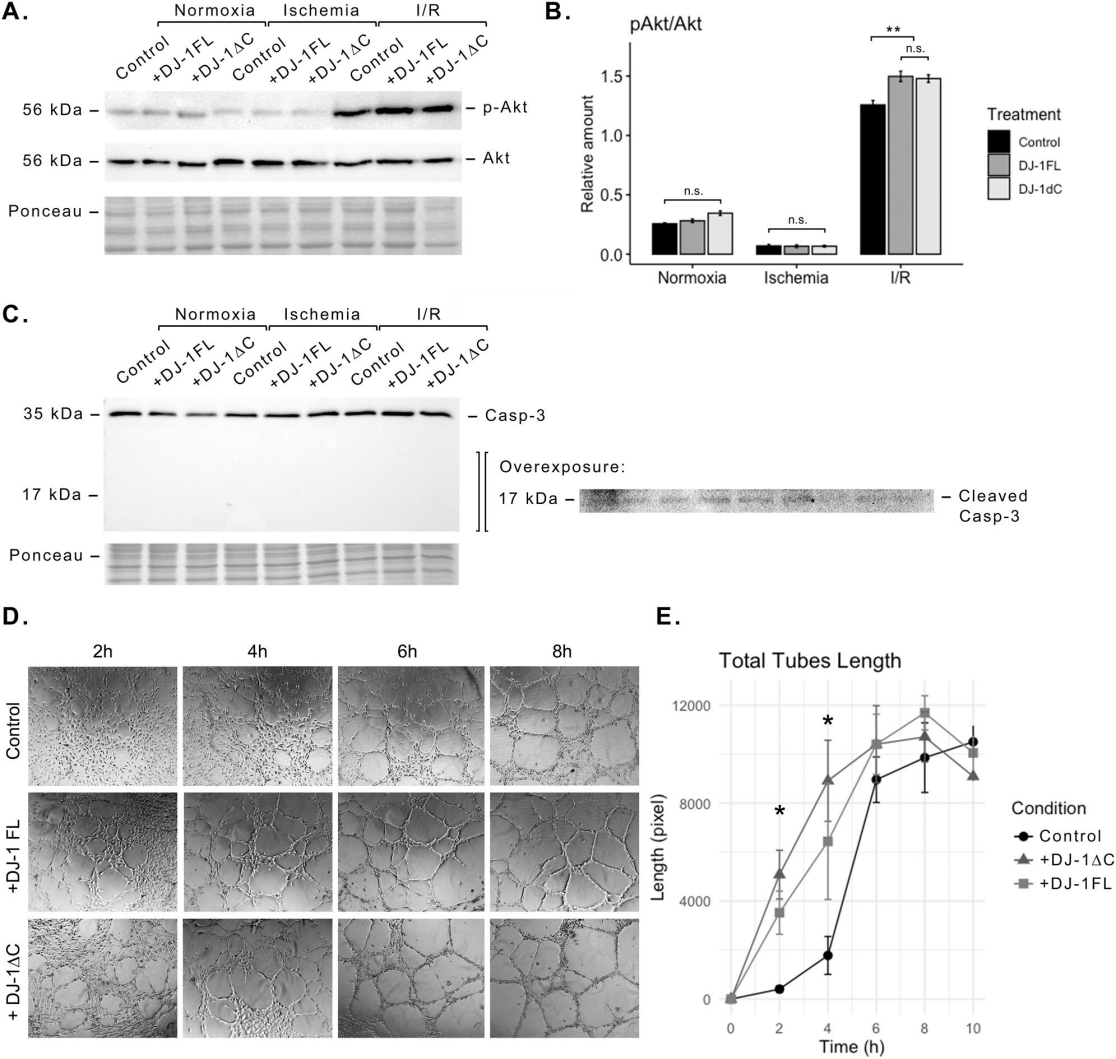
Effect of DJ-1 and DJ-1∆C ischemic exposure in signalling and in vitro angiogenesis. ECs cultures were exposed to either 1 h ischemia or normoxia, in the presence and the absence of exogenously administrated DJ-1 or DJ-1∆C at 100 nM, with and without a reperfusion period of 2 h, and both the phosphorylation status of Akt and the cleavage of Casp-3 were analysed upon cell lysates. (**A**) Representative western blot of phospho-Akt (Ser473) and total Akt in normoxia, ischemia, and I/R, in the presence and the absence of full-length DJ-1 or DJ-1∆C. (**B**) Akt relative phosphorylation status quantification (n = 4; **p < 0.01, *n.s.* not significant). (**C**) Representative western blot of Casp-3 in normoxia, ischemia, and I/R, in the presence and the absence of full-length DJ-1 or DJ-1∆C. No cleavage of Casp-3 was detectable in any condition, an overexposed acquisition of the 17 kDa surrounding region is included. (**D**) Representative acquisitions of tube-formation assay of ECs during reperfusion after the exposure to 1 h of ischemia, in the presence and the absence of exogenously administrated DJ-1 or DJ-1∆C at 100 nM. No exogenous DJ-1/DJ-1∆C were added in the reperfusion media. (**E**) Total tubes length quantification over reperfusion time (n = 3; *p < 0.05). *Casp-3* Caspase-3, *ECs* endothelial cells, *I/R* ischemia/reperfusion. Corresponding uncropped western blot acquisitions can be found in Supplemental Figs. 6 and 7.

Because the extracellular ATP generation through the ecATP-S has been reported to activate downstream signalling involved in cell survival via purinoreceptors^20^. We analysed the Akt phosphorylation status in normoxia, ischemia, and I/R, in the presence and the absence of exogenous full-length DJ-1 or DJ-1∆C (at 100 nM). While no differences were found between treated and untreated cultures in normoxia or ischemia, an enhanced Akt activation during reperfusion was found for the cultures treated with DJ-1 in either form (*p* < 0.01; Fig. 6A,B). Within the endothelial cell, Akt is involved in a number of processes ranging from cell survival and inhibition of apoptosis to angiogenesis^36–38^. In order to test whether the Akt activation at reperfusion after the exposure to DJ-1 is involved in cell survival or angiogenesis, we analysed both the cleavage of Casp-3, as a surrogate of apoptosis, and tube-formation capacity at reperfusion, as a surrogate of the angiogenic potential. As a result, no cleavage of Casp-3 was detectable in any condition (Fig. 6C), but a significantly faster formation of capillary-like structures during reperfusion was seen for the treated cultures (Fig. 6D,E). The sub-lethal nature of the assayed ischemic protocol, proven by the absence of Casp-3 cleavage, further confirms that all reported effects following DJ-1 administration are indeed dependent on DJ-1 and independent of cell death.

## Discussion

In the present study we characterized the endothelial dynamic of DJ-1 within the context of I/R injury, and explored its implications in the regulation of the ecATP-S activity following acute ischemia. We demonstrated that DJ-1 is actively cleaved and released by ECs, and depicted an autocrine effect upon the ecATP-S activity, extracellular ATP generation, and angiogenesis, in a human umbilical-vein endothelial cells (HUVEC) model of sub-lethal ischemia and I/R.

Ever since their first isolation by Jaffe *et* *al.* back in 1973^39^, to current days, HUVECs have become a valuable model for the in vitro study of vascular physiology and disease. As a non-immortalized human cellular model of ECs, HUVECs faithfully reproduce human ECs behaviour, and have been largely employed to study a broad array of biological processes and diseases^40^. Consequently, there are a number of standardized assays for the analysis of endothelial physiology and angiogenesis in HUVEC, facilitating the comprehension and reproducibility of results.

It is widely accepted that protein DJ-1 is implicated in cell survival following I/R and oxidative stress, as previously proven both in vitro and in vivo by loss-of-function models of myocardial infarction and stroke^34,41–47^. However, although many molecular functions have been attributed to DJ-1, there is not yet a consensus on its exact molecular function. DJ-1∆C arises from a 15 amino acid carboxyl-terminal deletion of DJ-1, resulting from a specific cleavage in response to mild oxidative stress, and have been purposed as a mechanism triggering its cytoprotective activity^24,33,48^. We evaluated the impact of I/R upon the DJ-1 reservoir of ECs, and found a significant and ischemia-dependent decline of the full-length form after reperfusion. This observation may be explained by protein degradation or release to the extracellular space. We further demonstrated that both forms were present in the cell secretome. Interestingly, DJ-1∆C secretion was found to be associated to ischemia, suggesting that DJ-1 is actively cleaved and released by ECs during ischemia. Moreover, we demonstrated DJ-1 to be secreted in a soluble form (i.e. not bound to EVs), as the ultracentrifugation of the cell culture media prior sample processing had no effect upon the detected extracellular DJ-1 and DJ-1∆C.

In order to avoid protein interference in the analysis of secretomes, we performed the secretion experiments under serum-free conditions, which may have affected the secretion process. Thus, two different controls were employed for ischemia and reperfusion, and so while both ischemia and first control underwent a transition from basal culture conditions to serum-free, both reperfusion and second control were kept in the absence of serum. Therefore, the observed differences in the DJ-1 secretion levels between the secretomes of the two controls arise from the acute cell stress induced by the transition from basal culture conditions to serum-free media.

Given the current consensus about a protective role for DJ-1, and the endothelial dynamic upon I/R injury^2,4,49^, the reported release of DJ-1 and DJ-1∆C may behave as a cell sensor for damage or as an autocrine/paracrine cell function modulator. Lacking of a conventional secretory signal peptide, DJ-1 has been previously suggested to be secreted through the autophagy-based unconventional secretion pathway, as proven by experiments with autophagy inhibitors and autophagy-related knockout models^50^. Being ischemia and I/R known stimuli to promote autophagy, this is could be a tentative and feasible mechanism. Yet, DJ-1 have been previously reported to be secreted under some pathologic conditions such as breast cancer^51^, Parkinson’s disease^52,53^ and stroke^54^, and a protective role upon ischemia^42^ and I/R^55^ have been proven for the extracellular form in neuronal cells. However, the mechanism by which extracellular DJ-1 confers protection remains to be clarified.

The ATP-synthase ectopic expression in the cell surface is now recognized for a number of cell types, and is known to display several functions ranging from angiogenesis to cholesterol uptake^6–8,21^. Being essentially a H^+^ channel, a role in the regulation of the intracellular pH is presumable, as shown by its inhibition with monoclonal antibodies targeted to the ectopic ATP-synthase, which resulted in a dysregulation of the intracellular pH^20^. This mechanism would be especially relevant in ischemia, which rapidly leads to acidosis. Indeed, the ecATP-S has been reported to highly increase its activity under chronic ischemic conditions^22^, and has been proposed as a mechanism of ischemia tolerance for ECs^56^. Additionally, the ecATP-S has been proposed to affect downstream signalling. Despite not yet elucidated, its location within the lipid rafts and caveolae allows it to modify the local ATP/ADP concentrations, potentially inducing purinergic signalling^9,11,21^. Treatments with monoclonal antibodies targeted to the ectopic ATP synthase were shown to antagonize Akt and Erk1/2 signalling, and to activate JNK and MAPK-p38, in ECs^20^. Also, the accumulation of extracellular ATP have been reported to protect endothelial barrier integrity following I/R injury^57^.

Here we report a great increase in the activity of the ecATP-S following acute ischemia in ECs, coinciding with DJ-1 and DJ-1∆C secretion. After 1 h of ischemia, control cultures exhibited nearly threefold increase in the extracellular ATP generation without changes in the relative amount of the cell-surface ATP-synthase. Such increase appeared to be dependent on DJ-1, as the inhibition of DJ-1 expression also inhibited the ecATP-S response to ischemia by ∼50%, and the administration of exogenous DJ-1 in either its full-length or cleaved form, maximized the effect. Noteworthy, the exogenous administration of DJ-1 in normoxia had no effect upon ecATP-S activity, possibly due to a lack of a proton gradient to drive its activity in the absence of acidosis. Hence, rather than activate the ecATP-S, extracellular DJ-1 seems to optimize its performance, as previously described in the mitochondria^32^. Furthermore, the exogenously administrated DJ-1 was proven to physically interact with the ATP-synthase in the same fashion seen for secretion. And so, interaction with ATP-synthase was found both under normoxia and ischemia for the full-length form of DJ-1, and exclusively under ischemia for the cleaved form. Such association was lost over the course of reperfusion in the absence of exogenous DJ-1.

Whilst ischemia represents a profound detrimental factor, with no other resolution than reperfusion, the rapid recovery of basal conditions paradoxically carries the potential to exacerbate damage itself in a process tightly correlated to the duration and severity of the ischemic insult^58^. Thus, despite being ischemia the most detrimental factor, the cell response to reperfusion directly impacts on the extent of damage^47^ In the context of ischemia and I/R, Akt has a number of positives effects as is the case of the inhibition of apoptosis and the promotion of angiogenesis^36–38^. Several reports have shown an activation of Akt during early reperfusion after ischemia^59^. Furthermore, the extent of this activation inversely correlates with damage, and thus Akt have been proposed as a central element of the I/R injury and the so called reperfusion injury salvage kinase (RISK) pathway^60,61^. Interestingly, while the extracellular ATP generation through the ecATP-S has been reported to activate Akt signalling via purinoreceptors^20^, no activation of Akt was seen during ischemia in any condition. However, the ischemic exposure to DJ-1 in either form resulted in a more pronounced Akt activation at reperfusion, which may reflect a physiologic adaptation or an enhanced viability of ECs at reperfusion, rather than a consequence of purinergic signalling. A faster formation of capillary-like structures during reperfusion was seen for the treated cultures in the tube-formation assay, suggesting an enhanced angiogenic potential. Moreover, these reported effects were seen to be independent of cell death, as proven by the Casp-3 cleavage analysis, where no induction of apoptosis was found in any tested condition. Previous reports have proven a role for the ecATP-S in angiogenesis, as the treatment with targeted antibodies show an inhibitory effect^62,63^. Whether this effect is indeed dependent on the extracellular ATP generation, a secondary effect of cell pH regulation, or another feature conditional to the ecATP-S, remain to be clarified. Altogether, the data here reported supports a role for the ecATP-S and DJ-1 in the preservation of endothelial homeostasis in ischemia and I/R.

## Methods

### Cell culture

Human umbilical vein endothelial cells (HUVEC) were cultured in gelatin (G1890; Sigma, Saint Louis, MI, USA) coated flasks with M-199 Hank’s medium (22350-029; Biological Industries, Beit-Haemek, Israel) containing 20% (v/v) FBS (04-007-1A; Thermo Fisher Scientific, Waltham, MA, USA), Endothelial Cells Growth Supplement (02-102; Millipore, Burlington, MA, USA), heparin (H3149; Sigma, Saint Louis, MI, USA), HEPES (15330-056; Thermo Fisher Scientific, Waltham, MA, USA), penicillin–streptomycin (15140-122; Thermo Fisher Scientific, Waltham, MA, USA), L-glutamine (25030-024; Thermo Fisher Scientific, Waltham, MA, USA), and pyruvate (11360-039; Thermo Fisher Scientific, Waltham, MA, USA), at 37 °C in 5% CO_2_ atmosphere. All experiments were performed between passage 4 and 8.

For knock-down studies cells were transfected with a *park7* siRNA (s22305; Thermo Fisher Scientific, Waltham, MA, USA) using the Amaxa Cell Line Nucleofector Kit V (VCA-1003; Lonza, Basilea, Switzerland) following manufacturer’s instructions. Both DJ-1 protein content and *park7* gene expression were assayed 72 h after transfection. All experiments were performed at 72 h post-transfection.

Cultures were treated with human recombinant full-length DJ-1 (MBS143125; MyBioSource, San Diego, CA, USA) or DJ-1∆C (made upon request; GenScript, Piscataway, NJ, USA) at 100 nM, when indicated.

### Ischemia and reperfusion model

Cell cultures were either subjected to in vitro ischemia or ischemia–reperfusion (I/R). In vitro ischemia was performed as previously described^64^, incubating cells in acidic PBS (pH = 6.4) under hypoxic atmosphere (1% O_2_). The culture of cells in growth medium and normoxic conditions, after a period of ischemia, is the modelling of reperfusion.

### Angiotube-formation assay

Tube-formation assay was performed as previously described^65^. Briefly, HUVEC were seeded in growth factor reduced MatriGel (Corning, New York, NY, USA) coated 48-well plates at a density of 30.000 cells/cm^2^, and let sit for 30 min. Then cultures were washed with PBS and subjected to 1 h in vitro ischemia in the presence and the absence of DJ-1 or DJ-1∆C at 100 nM. Immediately after, ischemia buffer was replaced with basal growth media (without DJ-1 and DJ-1∆C), and random acquisitions were taken every 2 h. Pictures were then analyzed in ImageJ (U.S. National Institute of Health, Bethesda, MD, USA).

### Secretome analysis

In order to avoid protein interference, analysis of the secretome was performed in serum-free conditions. HUVEC cultures were washed twice with PBS, and subjected to 1 h in vitro ischemia or kept in normoxia (1 h control). Immediately after, supernatants were collected and replaced by fresh medium. Cells were allowed to recover for 2 h. After such time, reperfusion and 2 h control supernatants were collected, and cells counted with a cell counter (Beckmann Coulter, Brea, California). Supernatants were centrifuged to discard detached cells and debris. Then, supernatants were either mixed with 5× radio-immune precipitation assay (RIPA) buffer supplemented with a protease inhibitior cocktail (05056489001; Roche Diagnostics, Mannheim, Germany), freeze, thawed, and sonicated, in order to lysate any extracellular vesicles (EVs) present, or ultracentrifuged for 1 h at 100.000 × *g*, to remove EVs. Appropriate volumes to normalize samples through the number of cells were precipitated with acetone at −20 °C overnight, re-solubilized with 1% SDS, dialyzed against PBS, and analysed by western blot.

### Western blot

Cell cultures were washed with PBS and lysed in ice-cold RIPA buffer supplemented with a protease inhibitior cocktail (05056489001; Roche Diagnostics, Mannheim, Germany). 10 µg of total protein were loaded and separated by SDS-PAGE with a Mini protean 3 system (Bio-Rad Laboratories, Hercules, CA, USA). Proteins were then transferred to a nitrocellulose membrane (1620115; Bio-Rad Laboratories, Hercules, CA, USA), and immediately stained with Ponceau S total protein staining (P3504; Sigma, Saint Louis, MI, USA), to normalize differences in the loaded protein amounts between lanes. Blots were then blocked with 5 % bovine serum albumin (MB04603; NZYTech, Lisboa, Portugal) diluted in TBS-Tween20 and incubated overnight with either mouse anti-DJ-1 (MCA-4H4, EnCor Biotechnology, Gainesville, FL, USA) at 1:4000, rabbit anti-ATP5B (MA5-32589, Invitrogen, Carlsbad, CA, USA) at 1:1000, rabbit anti-phospho-Akt Ser473 (4060; Cell Signaling Technology, Danvers, MA, USA) at 1:1000, rabbit anti-Akt (9272; Cell Signaling Technology, Danvers, MA, USA) at 1:1000, or rabbit anti-Casp-3 (9662; Cell Signaling Technology, Danvers, MA, USA) at 1:1000. Horseradish peroxidase-coupled rabbit anti-mouse IgG secondary antibody or goat anti-rabbit IgG (P0260/P0448; Dako, Santa Clara, CA, USA) were used to detect primary antibodies together with SuperSignal reagent (34076; Thermo Fisher Scientific, Waltham, MA, USA). All images were acquired with a ChemiDoc XRS system (Bio-Rad Laboratories, Hercules, CA, USA).

### Real-time qPCR

Cells were washed with PBS, and total RNA was isolated with the RNeasy Mini Kit (74106; Quiagen, Hilden, Germany) according to the manufacturer’s instructions. Transcript levels were analysed by real-time quantitative polymerase chain reaction (qPCR) with on-demand TaqMan assays (DJ-1: Hs00994893_g1; Thermo Fisher Scientific, Waltham, MA, USA). rRNA-18S was used as an endogenous control (Hs99999901_s1; Thermo Fisher Scientific, Waltham, MA, USA). Taq-man real-time qPCR was performed as previously described^66^.

### Ectopic ATP-synthase activity

ecATP-S activity was analysed as previously described^19^. Briefly, cells were washed once in HEPES buffer (10 mM HEPES, 150 mM NaCl), and incubated for 5 min in HEPES buffer supplemented with 2 mM MgCl_2_. Extracellular ATP generation was then initiated with the addition of HEPES buffer supplemented with 2 mM MgCl_2_, 20 mM KH_2_PO_4_ and 200 µM ADP. After 20 s of reaction, supernatants were collected in EDTA containing tubes at 5 mM final concentration and centrifuged at 300x*g* 10 min at 4 °C to discard detached cells. ATP concentration was then assayed in supernatants with a luminescent ATP detection assay kit (ab113849; Abcam, Cambridge, United Kingdom) following manufacturer’s instructions. After supernatants collection, cells were immediately lysed and protein amounts were quantified to normalize between wells.

### Immunostaining

After indicated treatments, cells were washed with PBS and fixed for 15 min with 4% paraformaldehyde at room temperature. No permeabilization step was performed. Unspecific bindings were then blocked with 1% bovine serum albumin (MB04603; NZYTech, Lisbon, Portugal) diluted in PBS, for 15 min two times. Then cells were rinsed twice, incubated for 30 min with Image-IT FX signal enhancer (136,933; Invitrogen, Carlsbad, CA, USA), washed again and incubated with rabbit anti-ATP5B (MA5-32589, Invitrogen, Carlsbad, CA, USA) at 1:200, 1 h at room temperature. Cells were then thoroughly rinsed and incubated for 1 h with Alexa Fluor 488 coupled donkey anti-rabbit (A21206; Invitrogen, Carlsbad, CA, USA) at 1:100, counterstained with Hoechst 33342 (H3570; Thermo Fischer Scientific, Waltham, MA, USA) at 1 µg/mL, and mounted in Prolong Gold (P36931; Invitrogen, Carlsbad, CA, USA). Five random images per sample were then acquired using a Leica TCS SP5 laser scanning confocal microscope (Leica microsystems, Wetzlar, Germany). Between 10 and 20 acquisitions per field were taken with a z-stack step size of 0.5 µm, in order to capture the whole sample volume. Maximum projections were then analysed with ImageJ (U.S. National Institute of Health, Bethesda, MD, USA).

### Immunoprecipitation

Cell cultures were lysed in ice-cold (RIPA) buffer supplemented with a protease inhibitior cocktail (05056489001; Roche Diagnostics, Mannheim, Germany). Lysates were then incubated overnight with rabbit anti-ATP5B (MA5-32589, Invitrogen, Carlsbad, CA, USA) at 1:100, with gentle rocking at 4 °C. Protein G Sepharose beads (17-5280-04; GE Healthcare, Chicago, IL, USA) were used for precipitation according to the instructions of the manufacturer.

### Statistical analysis

Normality was assessed with the Shapiro–Wilk method. When normality could be assumed, statistical differences between groups were analysed by two-tailed t-test (for comparisons between two groups), one-way ANOVA (for multiple groups) or two-way ANOVA (for multiple groups and two factors). Tukey’s Honestly Significant Difference (HSD) *post hoc* test was performed to correct significance for multiple-comparisons. Kruskal–Wallis rank sum test was performed when normality could not be assumed. Data is presented as mean ± SEM. All the analyses were performed in RStudio (RStudio, Boston, MA, USA).

## Supplementary Information

Supplementary Figures.

## Data Availability

The datasets used and/or analysed during the current study are available from the corresponding author on reasonable request.
