# Supplementary material for: DJ-1 interacts with the ectopic ATP-synthase in endothelial cells during acute ischemia and reperfusion
Source: Sci Rep. 2022 Jul 26;12:12753. doi: 10.1038/s41598-022-16998-3 (PMC9325725; doi:10.1038/s41598-022-16998-3)

Supplemental Figure 1

Time course analysis of endothelial DJ-1ΔC at reperfusion. HUVEC cultures were subjected to 1h of *in vitro* ischemia followed by reperfusion, and relative DJ-1ΔC content was assayed at different time-points in cell lysates by western blot. **A.** Representative western blot of DJ-1 and DJ-1ΔC protein content over reperfusion. **B.** Quantification of intracellular relative DJ-1ΔC content over reperfusion. Shaded area indicates ischemia. HUVEC, Human umbilical-vein endothelial cells. Uncropped western blots acquisitions can be found in Supplemental Figure 8.

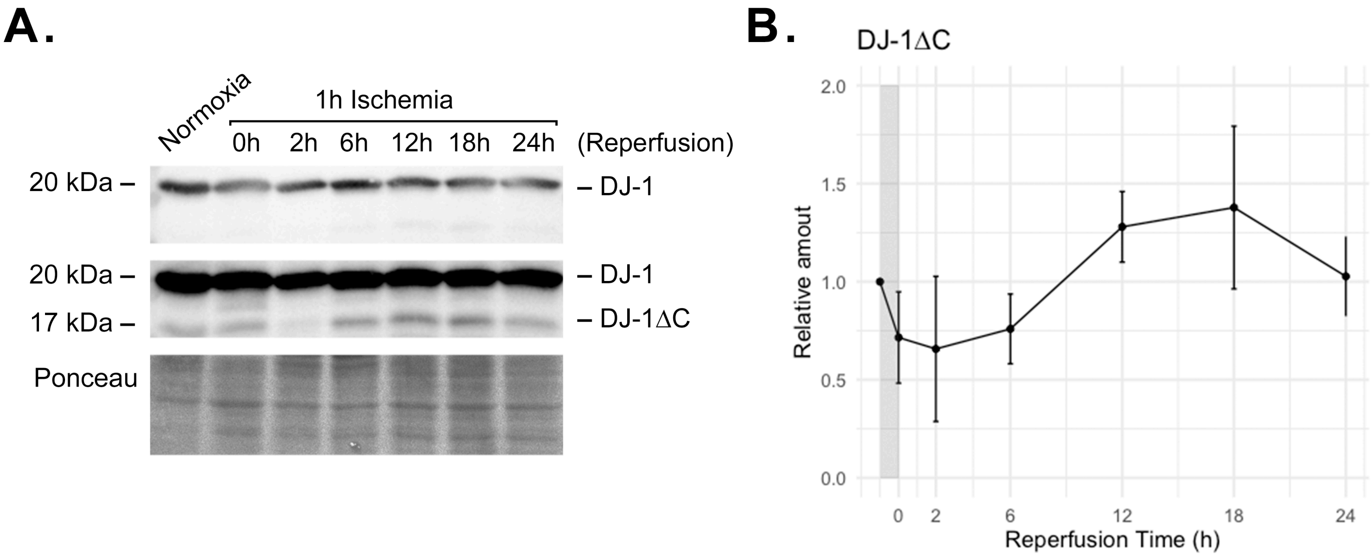

Supplemental Figure 2

Endothelial DJ-1 content declines during I/R injury - supporting gels. ECs cultures were subjected to either 30 min or 1 h of in vitro ischemia followed by 2 or 22 hours of reperfusion, and DJ-1 content was analysed by western blot. **A.** Uncropped western blot acquisition for DJ-1. **B.** Uncropped western blot overexposure for DJ-1ΔC. **C.** Corresponding uncropped Ponceau-S total protein staining acquisition. ECs, Endothelial cells; I/R, Ischemia-reperfusion.

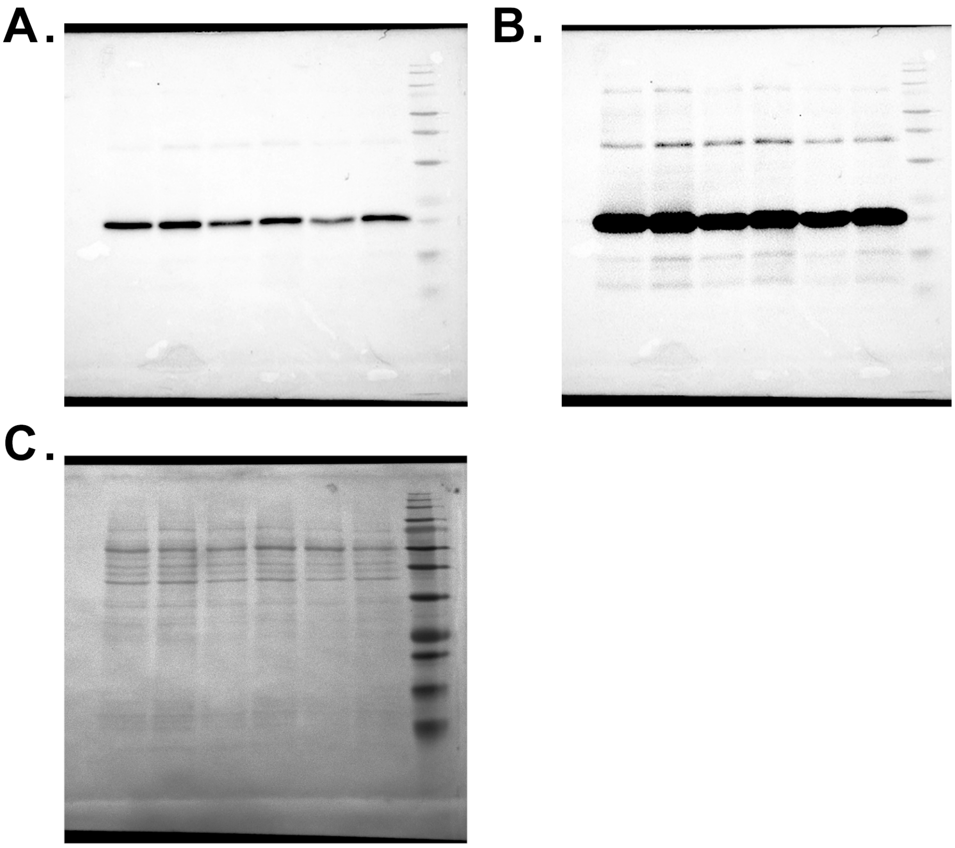

Supplemental Figure 3

In vitro I/R promotes DJ-1 and DJ-1ΔC endothelial secretion - supporting gels. ECs cultures were subjected to 1 h of in vitro ischemia followed by 2 h of reperfusion, and supernatants from ischemia, reperfusion, and control cultures, were proved for DJ-1. **A.** Uncropped western blot acquisition for secreted DJ-1. **B.** Uncropped western blot acquisition for DJ-1, before and after ultracentrifugation for 1h at 100.000*g* (alternate samples). ECs, Endothelial cells.

A.

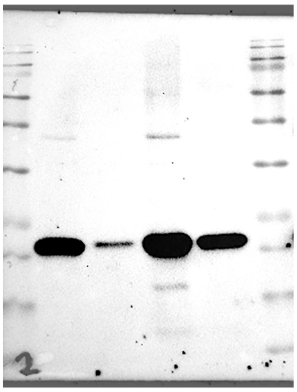

B.

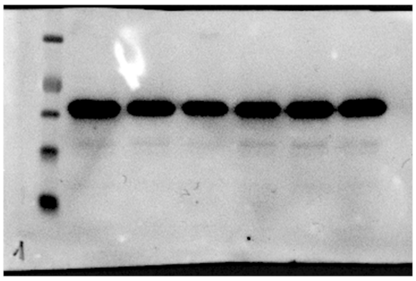

Supplemental Figure 4

Extracellular ATP generation after ischemia is dependent on DJ-1 - supporting gels. ECs cultures were transfected with a siRNA targeted to DJ-1, and subjected to 1 h of *in vitro* ischemia. Extracellular ATP generation was then evaluated. **A.** Uncropped western blot acquisition for DJ-1 for transfected and control cultures (alternate samples). **B.** Corresponding uncropped Ponceau-S total protein staining acquisition. ECs, Endothelial cells.

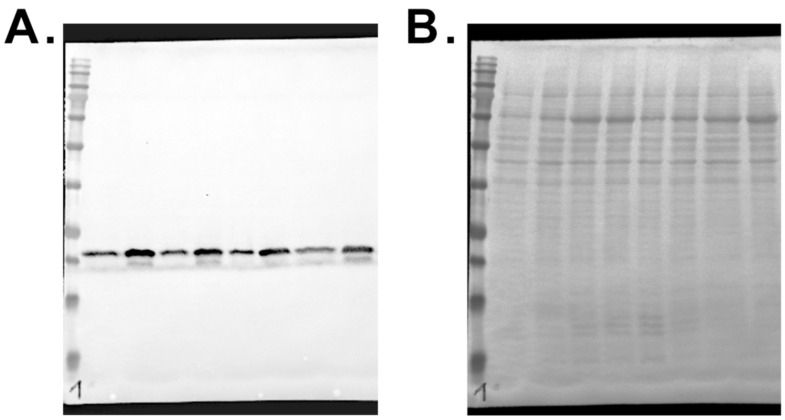

Supplemental Figure 5

ATP-synthase co-immunoprecipitation with exogenous DJ-1 and DJ-1ΔC in ischemia and I/R - supporting gels. ECs cultures were exposed to either 1h of ischemia or normoxia, in the presence and the absence of exogenously administrated with DJ-1 or DJ-1ΔC at 100 nM, with and without a reperfusion period of 2h and 24h in the absence of exogenous DJ-1/DJ-1ΔC. After indicated treatment, cells were thoroughly rinsed, lysed, and immunoprecipitated against ATP-synthase (ATP5B). Both total lysates (**A**-**D**) and IP-captures (**E**-**H**) were probed for ATP5B (**A**,**C**,**E**, and **G**) and DJ-1 (**B**,**D**,**F**, and **H**). Corresponding uncropped western blot acquisitions are presented. Membranes were split in two before incubation. ECs, Endothelial cells; IP, immunoprecipitate; I/R, ischemia/reperfusion.

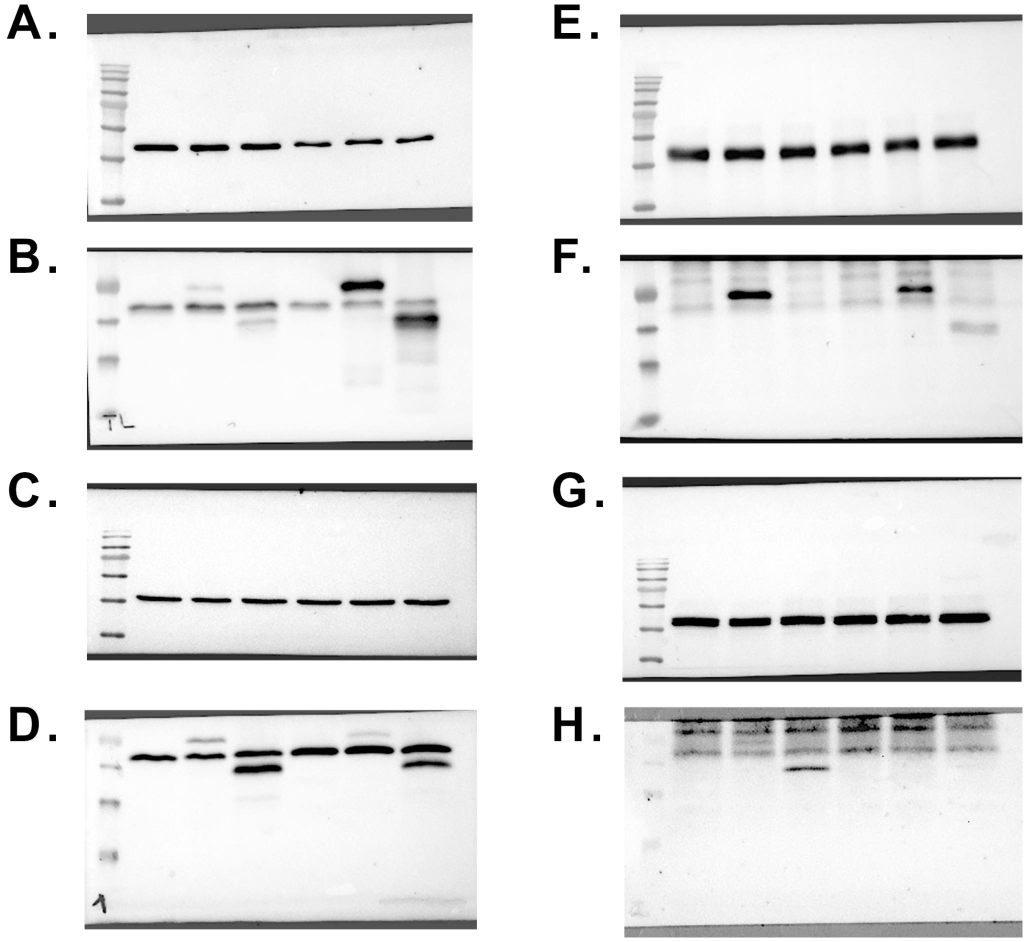

Supplemental Figure 6

Effect of DJ-1 and DJ-1ΔC ischemic exposure in Akt signalling - supporting gels. ECs cultures were exposed to either 1h of ischemia or normoxia, in the presence and the absence of exogenously administrated DJ-1 or DJ-1ΔC at 100 nM, with and without a reperfusion period of 2h, and the phosphorylation status of Akt was analysed upon cell lysates. **A.** Uncropped western blot acquisition for phospho-Akt (Ser473) analysis. **B.** Uncropped western blot acquisition for total Akt analysis. **C.** Corresponding uncropped Ponceau-S total protein staining acquisition. ECs, Endothelial cells; I/R, Ischemia/reperfusion.

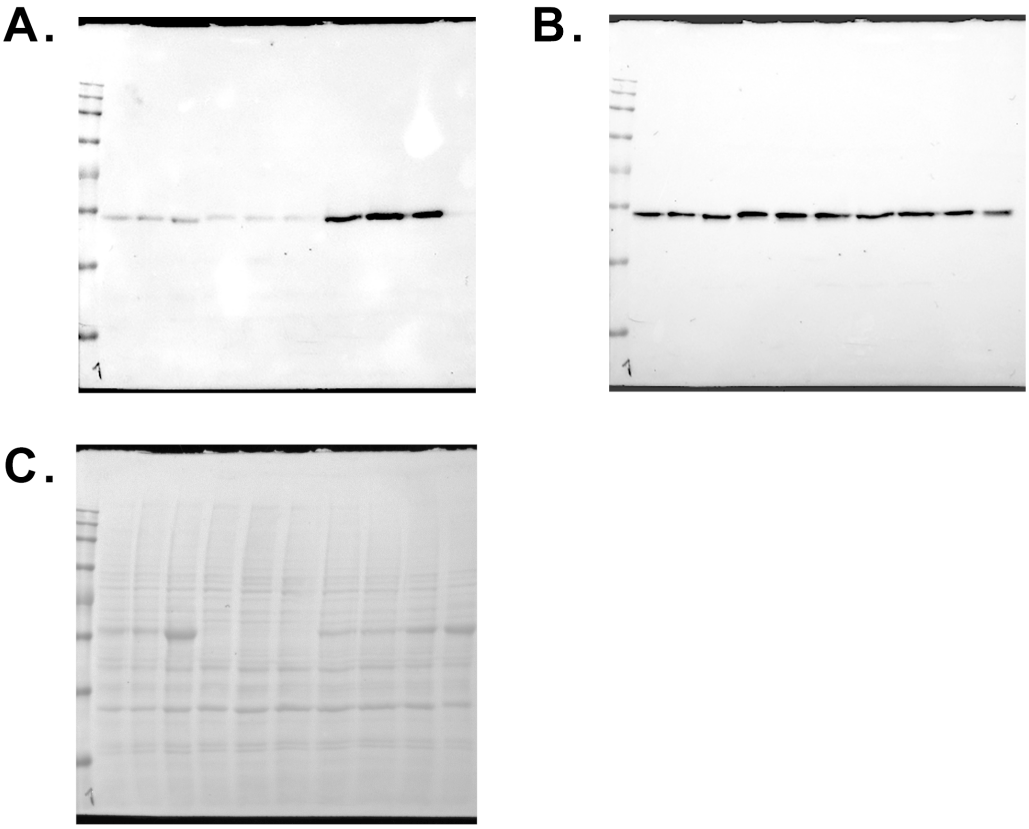

Supplemental Figure 7

Effect of DJ-1 and DJ-1ΔC ischemic exposure in Casp-3 cleavage - supporting gels. ECs cultures were exposed to either 1h of ischemia or normoxia, in the presence and the absence of exogenously administrated DJ-1 or DJ-1ΔC at 100 nM, with and without a reperfusion period of 2h, and the cleavage of Casp-3 was analysed upon cell lysates. **A.** Uncropped western blot acquisition for Casp-3 analysis, and overexposure of the low molecular weight section of the membrane. **B.** Corresponding uncropped Ponceau-S total protein staining acquisition. Casp-3, Caspase-3; ECs, Endothelial cells; I/R, Ischemia/reperfusion.

A.

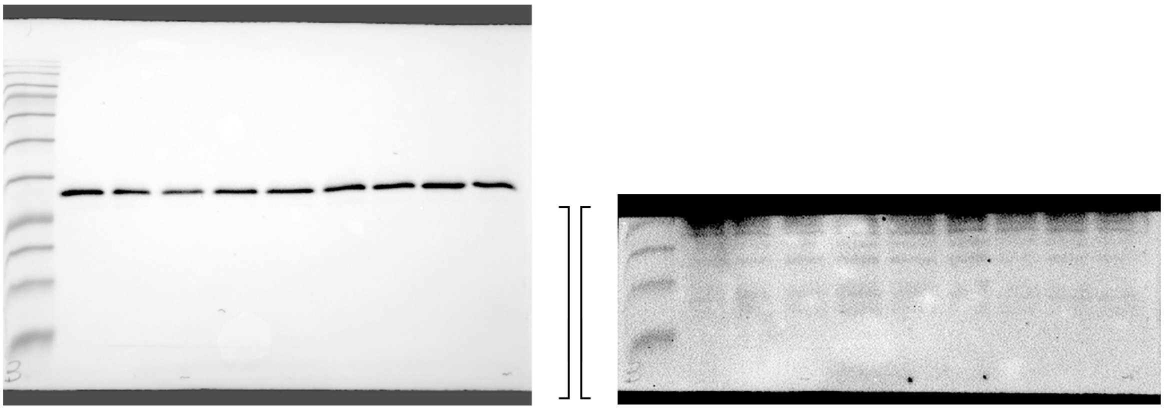

B.

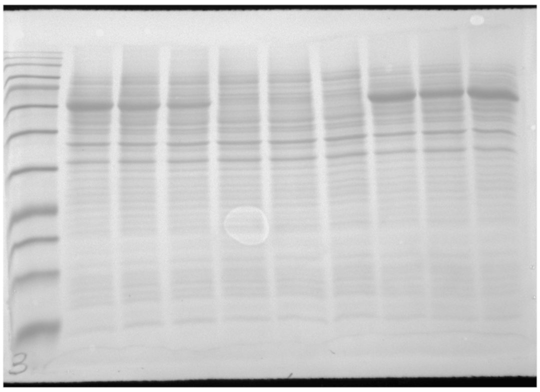

Supplemental Figure 8

Time course analysis of endothelial DJ-1ΔC at reperfusion - supporting gels. HUVEC cultures were subjected to 1h of *in vitro* ischemia followed by reperfusion, and relative DJ-1ΔC content was assayed at different time-points in cell lysates by western blot. **A.** Uncropped western blot acquisition for DJ-1 analysis. **B.** Uncropped western blot overexposure for DJ-1ΔC analysis. **C.** Corresponding uncropped Ponceau-S total protein staining acquisition. ECs, Endothelial cells; I/R, Ischemia-reperfusion.

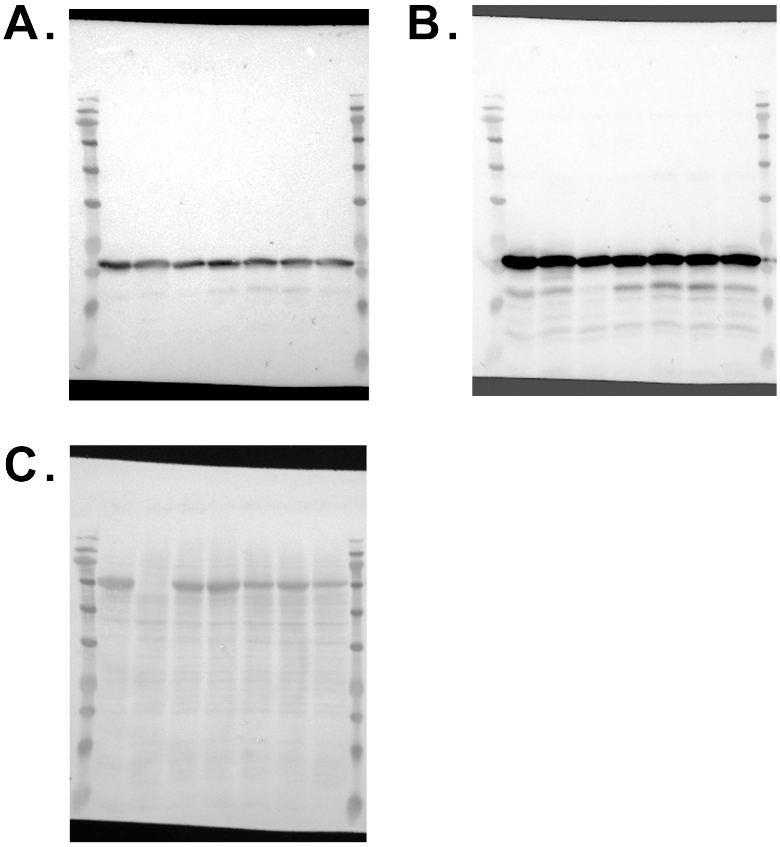

Supplement: Supplementary file 1 — Supplementary Figures. [file 41598_2022_16998_MOESM1_ESM.pdf]
